# Molecular Characterizations of the Coagulase-Negative *Staphylococci* Species Causing Urinary Tract Infection in Tanzania: A Laboratory-Based Cross-Sectional Study

**DOI:** 10.3390/pathogens12020180

**Published:** 2023-01-24

**Authors:** Shukrani Phillip, Martha F. Mushi, Arun Gonzales Decano, Jeremiah Seni, Blandina T. Mmbaga, Happiness Kumburu, Eveline T. Konje, Joseph R. Mwanga, Benson R. Kidenya, Betrand Msemwa, Stephen Gillespie, Antonio Maldonado-Barragan, Alison Sandeman, Wilber Sabiti, Mathew T. G. Holden, Stephen E. Mshana

**Affiliations:** 1 Institute of Allied Health Sciences, Ruaha Catholic University, Iringa P.O. Box 774, Tanzania; 2 Department of Microbiology and Immunology, Weill Bugando School of Medicine, Catholic University of Health and Allied Sciences, Mwanza P.O. Box 1464, Tanzania; 3 School of Medicine, University of St Andrews, St Andrews KY16 9AJ, UK; 4 Kilimanjaro Clinical Research Institute, Moshi P.O. Box 2236, Tanzania; 5 Department of Epidemiology and Biostatistics, Catholic University of Health and Allied Sciences, Bugando, Mwanza P.O. Box 1464, Tanzania; 6 School of Public Health, Catholic University of Health and Allied Sciences, Bugando, Mwanza P.O. Box 1464, Tanzania; 7 Department of Biochemistry and Molecular Biology, Weill Bugando School of Medicine, Catholic University of Health and Allied Sciences, Mwanza P.O. Box 1464, Tanzania

**Keywords:** *S. epidermidis*, *S. haemolyticus*, icaC virulence genes, genes for AMR

## Abstract

Background: There is a growing body of evidence on the potential involvement of coagulase-negative Staphylococci (CoNS) in causing urinary tract infections (UTIs). The aim of this study was to delineate virulence potential, antimicrobial resistance genes, and sequence types of CoNS isolated from patients with UTI symptoms and pyuria in Tanzania. Methods: CoNS from patients with UTI symptoms and more than 125 leucocytes/μL were retrieved, subcultured, and whole-genome sequenced. Results: Out of 65 CoNS isolates, 8 species of CoNS were identified; *Staphylococcus haemolyticus*, *n* = 27 (41.5%), and *Staphylococcus epidermidis*, *n* = 24 (36.9%), were predominant. The majority of *S. haemolyticus* were sequence type (ST) 30, with 8 new ST138-145 reported, while the majority of *S. epidermidis* were typed as ST490 with 7 new ST1184-1190 reported. Sixty isolates (92.3%) had either one or multiple antimicrobial resistance genes. The most frequently detected resistance genes were 53 (21%) *dfr*G, 32 (12.9%) *bla*Z, and 26 (10.5%) mecA genes conferring resistance to trimethoprim, penicillin, and methicillin, respectively. Out of 65 isolates, 59 (90.8%) had virulence genes associated with UTI, with a predominance of the *ica*C 47 (46.5%) and *ica*A 14 (13.9%) genes. **Conclusion:** *S. haemolyticus* and *S. epidermidis* harboring *ica*C, *dfr*G, *bla*Z, and *mec*A genes were the predominant CoNS causing UTI in Tanzania. Laboratories should carefully interpret the significant bacteriuria due to CoNS in relation to UTI symptoms and pyuria before labeling them as contaminants. Follow-up studies to document the outcome of the treated patients is needed to add more evidence that CoNS are UTI pathogens.

## 1. Introduction

Globally, urinary tract infection (UTI) affects about 150 million patients annually with a recurrence rate of 27% among women within 6 months of the first episode [1]. In Tanzania, UTI is reported to affect about 2 in every 10 pregnant women [2,3] and 2–3 in every 10 children [4,5,6] annually. Given the decline in malarial prevalence and incidence, UTI is the third most common type of illness affecting children of five years old and above and second most common illness affecting patients visiting outpatient departments (OPDs) in Tanzania [6]. Gram-negative bacteria of the order Enterobacterales have commonly been implicated in causing UTI [7,8]. Furthermore, studies have found associations between UTI and coagulase-negative staphylococci (CoNS) [8,9,10]. The CoNS reported to be involved in causing UTIs include: *Staphylococcus haemolyticus*, *Staphylococcus saprophyticus*, *Staphylococcus epidermidis*, *Staphylococcus hominis*, *Staphylococcus xylosus*, *Staphylococcus simulans*, and *Staphylococcus cohnii* [8,11,12]. CoNS possess genes encoding virulence factors associated with causation of UTI such as: biofilm formation (icaABC, aap, bhp, atlE, fbe, and embp) [13], hemolysis of red cells (hlg and hla), and adhesion and cell wall-anchored proteins (fnbA, ebpS and sasA, sasF, sasH) [14,15,16,17].

CoNS species have been documented to have diverse sequence types implicated in different clinical conditions. For example, *S. epidermidis* ST1, ST2, ST5, and ST215 have been associated with both hospital-acquired and community-acquired UTI [18]. *S. lugdunensis* ST3, ST2, and ST1 have been associated with human clinical isolates (skin or soft tissue, osteoarticular, blood, and material device isolates) [19]. *S. epidermidis* ST2, ST54, ST28, ST59, ST490, and ST596 have been frequently isolated from blood [20].

In Tanzania, significant bacteriuria cases due to CoNS have been reported to range from 6.2% to 16.7% among women with diabetic and febrile children [21,22], without delineating the specific species involved and their antimicrobial resistance or virulence genes determinants. A recent study in Tanzania using VITEK MS identified *S. haemolyticus* to be the second most common Gram-positive uropathogen causing community UTIs [23].

In most clinical microbiology laboratories, CoNS are regarded as skin contaminants [24,25]. This practice might have contributed to the mismanagement of patients. In line with this gap, the current study used whole-genome sequencing (WGS), to determine species, sequence types, virulence potential, and antimicrobial resistance genes of CoNS isolated from patients who were clinically and microbiologically confirmed to have UTI caused by CoNS.

## 2. Results

### 2.1. Bacteria Speciation and Distribution of CoNS Species from Urine Samples

**Figure 1:**
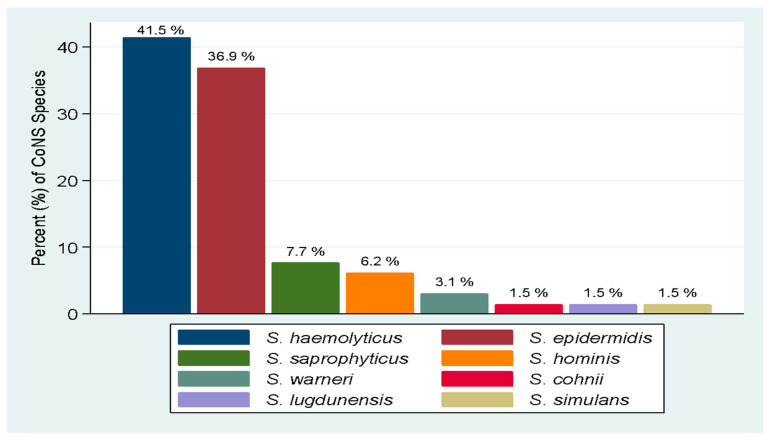
Distribution of CoNS species causing UTI.

Of the 79 isolates recovered, 65 (82.3%) were confirmed to be CoNS species by WGS, and were identified to 8 different CoNS species (Figure 1). The predominant CoNS species detected were *S. haemolyticus* *n* = 27 (41.5%) and *S. epidermidis* *n* = 24 (36.9%).

### 2.2. Multilocus Sequence Types of CoNS Species Causing UTI

Out of 65 CoNS isolates, 24 (36.9%) revealed 11 different sequence types (STs) in the first attempt. Out of the 27 *S. haemolyticus* isolates, 18 were assigned to 6 different STs predominated by ST30, and 9 isolates were assigned to 8 new ST138-145.

Among the 24 *S. epidermidis* isolates, 9 were assigned to 3 different STs predominated by ST490, and 7 isolates were assigned to 7 new ST 1184-1190 while 8 isolates with mutation had unknown ST and have been submitted to the *S. epidermidis* pubMLST database (https://pubmlst.org/organisms/staphylococcus-epidermidis; accessed on 10 January 2023) (Table 1). Majority of the isolates with unknown ST had three-point mutation in arcC_8, Supplementary Material File S1.

### 2.3. Virulence Genes of CoNS Species Causing UTI

A total of 5 different virulence genes associated with UTIs were identified in 59 (*n* = 65, 90.8%) isolates (Table 2). Genes responsible for the formation of polysaccharide intercellular adhesin produced by a gene cluster at the intercellular adhesion (*ica*) locus were predominantly detected, led by icaC, which was present in 47 isolates (46.5%). The highest virulence genes combination was 5 (*ica*A, *ica*B, *ica*C, *ica*D, fbe), identified in 15 isolates. A total of 6 CoNS isolates had no known virulence genes associated with UTIs.

### 2.4. Antimicrobial Resistance Genes Identified among CoNS Species

Out of 65 CoNS isolates, 60 (92.3%) had one or multiple genes coding for antimicrobial resistance (AMR). Most of the resistance genes were identified among *S. haemolyticus* and *S. epidermidis* isolates (Table 3). The most frequently identified gene was *dfr*G (*n* = 53, 21.4%), which confers resistance to trimethoprim. Other frequently identified genes included *bla*Z (*n* = 32, 12.9%) and *mec*A (*n* = 26, 10.5%), which confer resistance to penicillin and methicillin, respectively. Totals of 24 (88.9%), 21 (77.8%), and 21 (77.8%) *S. haemolyticus* had *dfr*G, *bla*Z, and *mec*A genes, respectively, while *dfr*G was identified in 22 (91.7%) isolates of *Staphylococcus epidermidis*.

## 3. Discussion

Coagulase-negative staphylococci (CoNS) are usually opportunistic pathogens, but have become important pathogens in clinical microbiology laboratories associated with various clinical conditions, such as UTI, skin and soft tissue infections, septicemia, and osteoarticular infections [8,19,26,27,28,29]. This study documents that the varieties of CoNS species causing UTI were predominantly *S. haemolyticus* and *S. epidermidis*. The CoNS species investigated were endowed with virulence genes, mainly icaC, which has been reported to facilitate the pathogenesis of UTI. Furthermore, these CoNS isolates harbored AMR genes with the predominance of *dfr*G, *bla*Z, and *mec*A genes.

As reported recently by Vitus et al. [23], *S. haemolyticus* was the most predominant CoNS detected as a uropathogen in the current study. *S. haemolyticus* is among the normal skin microbiota commonly found in the perineum and inguinal area, making it easy for them easy to ascend and cause UTI [30,31]. Furthermore, *S. haemolyticus* is known to harbor over 82 insertion sequences [32], antibiotic-resistance genes, and some virulence factors, which confer adaptability to different environments and highlight unusual plasticity [30]. The findings also concur with other studies outside Tanzania that reported 49.4% of Gram-positive bacteria causing UTI were *Staphylococcus haemolyticus* [33,34]. These findings indicate the possibility of *S. haemolyticus* being among the leading uropathogenic CoNS.

The second most identified species was *S. epidermidis* (39.6%), which was more common compared to 15.2% and 9.14% documented in Libya and China, respectively [11,34]. The differences across the three countries may indicate varying geographical conditions that favor or inhibit their growth. *S. epidermidis* is one of the most common skin opportunistic pathogens. Other CoNS species identified in the current study included *S. saprophyticus*, *S. hominis*, *S. lugdunensis*, *S. simulans, S. warneri*, and *S. cohnii*. These isolates have also been isolated from symptomatic UTI patients in previous studies [11,34,35]. *S. saprophyticus* has been widely documented as one of the commonest causes of UTI among CoNS species; to our surprise, this pathogen was not commonly detected in the current study despite the study population including young women, emphasizing the need to update our epidemiological knowledge, since *S. saprophyticus* has been reported to account for 5% to 15% of uncomplicated lower UTI cases in young women [36,37,38].

Multilocus sequence typing can discriminate between strains of the same species irrespective of their clinical conditions and sites [39]. *S. haemolyticus* was assigned to five different sequence types (ST30, ST1, ST38, ST49, and ST56) including three ST (ST30, ST1, ST38) previously detected from different clinical samples such as eyes, blood, pus, and sputum [40]. Furthermore, 37% of *S. haemolyticus* were assigned to new ST138-145, indicating difference in epidemiological distribution of these isolates between developed and developing countries because most of known ST were from studies in developed countries. Additionally, in the current study, a total of 33.3% of *S. epidermidis* were not assigned to known STs. Three types of STs (ST150, ST329, and ST490) assigned to *S. epidermidis* were detected, as in previous studies but from other human infections apart from UTI [20,41], 7 new ST1184-1190 have been reported for the first time in this study indicating population epidemiological differences of *S. epeidermidis*. ST3 *S. lugdunensis* detected in the current study was previously documented from skin or soft tissue, osteoarticular, and blood isolates [19]. The diversity of the STs in this study and other studies elsewhere indicate geographical variations that may dictate inheritance; hence, connoting multiple sources or niches of CoNS.

Studies have reported virulence genes such as *ica*ADBC, hla, hla_yidD, hld, and hlb to be implicated in UTI [14,15,17]. In a current study, 90.8% of CoNS were found to harbor UTI virulence genes. This is relatively high compared to previous study reported in India, where 56.1% of CoNS possessed either one or multiple virulence genes [42]. The difference could be explained by the selection of patients, whereby our patients had clinical infections (we only included patients with signs and symptoms and pyuria) contrasting the previous study that had different clinical samples, including exudates, urine, blood, endotracheal, catheter tips, and sputum. The production of poly-N-acetylglucosamine (PNAG) is crucial for biofilm formation in CoNS species and is coded in the *ica*ADBC genes cluster. Biofilm formation protects these bacteria against the antibacterial drugs and the immune system defenses [43]. In the current study, 95.8% of *S. epidermidis* were detected to have genes coding for biofilm formations (either of *ica*C, *ica*D, *ica*A, and *ica*B single or in combination). The proportion of biofilm encoding genes detected in the current study is much higher than 22.5% reported by Solati et al. [44].

The fbe gene, encoding for fibrinogen binding protein, mediates initial attachment to cell walls during biofilm formation was detected in 12.9% among CoNS, which was relatively lower compared to a 20% and 40% among strong and moderate biofilm forming CoNS, respectively, as reported previously [42].

The *dfr*G was the most frequently identified (21.4%) AMR determinant gene in the collection of CoNS isolates in the current study. The *dfr*G gene encodes for a dihydrofolate reductase that confers resistance to trimethoprim. The resistance to trimethoprim is escalating over the time; this might be propagated by overuse of this antibiotic as it is cheap, highly accessible over the counter in our settings, and has been extensively used as a prophylaxis against *Pneumocystis jirovecii* pneumonia among HIV patients [45].

The *bla*Z gene, which is usually found in plasmid and confers resistance to penicillin, was identified in 49.2% of isolates, which is low compared to 86.8% reported among CoNS in India [46]. The *bla*Z gene encodes for a β-lactamase that is synthesized when staphylococci are exposed to β-lactam antibiotics, thus cleaving the β-lactam ring, rendering the penicillin inactive [47].

The current study documents the prevalence of the *mec*A gene (encoding for an altered penicillin-binding protein (PBP 2a), which confers resistance to methicillin [48]) to be 40% among CoNS isolates causing UTI. This is low compared to 70.7% documented by Shrestha et al. [49] in Nepal. The difference in the findings from these two studies might be due to the type of patients studied; Shrestha et al. used patients in intensive care units who had received several courses of antibiotics from primary care hospitals while the majority of patients in the current study are from community-acquired UTIs.

The findings reported from this study emphasize the need to strengthen the diagnosis capacity in microbiology laboratory, especially in lower resource settings, to include tests that can correctly identify CoNS species. Additionally, in patients with clinical signs and symptoms, the study findings indicate the need to consider them for treatment and not neglect them as skin contaminant due to their virulence potential elaborated here. Regardless of all their usefulness, the study findings are limited by the small number of isolates involved, which could lead to the absence of some important findings such as *S. saprophyticus.*

## 4. Materials and Methods

### 4.1. Study Design and Area

This was a laboratory based cross sectional study designed to characterize of CoNS isolates collected by HATUA project 2018 to 2020, from three sites in Tanzania i.e., Mwanza, Kilimanjaro and Mbeya.

For this study, the laboratory work was conducted from February 2021 to August 2021 in three different laboratories at the Microbiology Research Laboratory of the Catholic University Health and Allied Sciences—Bugando, Mwanza, and Kilimanjaro Clinical Research Institute (KCRI) Biotechnology Laboratory, Moshi-Kilimanjaro. Genome sequencing of isolates was carried out at MicrobesNG, University of Birmingham, UK.

### 4.2. Study Population

Isolates collected in this study were part of the HATUA study [50]. Archived CoNS isolates from patients with UTI-like symptoms, pyuria of above 125 leucocytes/μL, and significant bacteriuria obtained as described in the HATUA protocol [50]. In Tanzania, the HATUA project recruited patients with clinical diagnosis of UTI from 10 different health facilities in Mwanza, Kilimanjaro, and Mbeya. Clinically suspected UTI patients were those with signs/symptoms (e.g., fever, burning/irritation during urination, dysuria, pyuria) and microbiologically confirmed were those with significant bacterial growth of >10^5^ CFU/mL in quantitative urine culture [50].

### 4.3. Sample Size and Isolate Recovery

The study used all CoNS isolates that could be recovered from the HATUA biorepository at CUHAS. A total of 101 CoNS isolates previously identified during HATUA were retrieved from the biorepository. On subsequent subculture onto 5% sheep blood agar (SBA) and incubated for 18 to 24 h aerobically at 37 °C, a total of *n* = 79 isolates were fully recovered. Recovered isolates were (*n* = 79) transported in sterile microbiological transport swab containing Stuart medium (Guangzhou Improved Medical Instruments Co., Ltd., Guangzhou, China) to MicrobesNG, University of Birmingham, UK (*n* = 59) and KCRI Biotechnology Laboratory Moshi-Tanzania (*n* = 20), where subculture was performed, followed by DNA extraction and WGS.

### 4.4. DNA Extraction and Library Preparation

Pure cultures of each strain were grown in plates or broth. Cells were harvested and suspended in a tube with cryopreservative (MicrobankTM, Pro-Lab Diagnostics UK, London, UK) or with DNA/RNA Shield (Zymo Research, Irvine, CA, USA) following MicrobesNG strain submission procedures.

First, 5 to 40 μL of the suspension were lysed with 120 μL of TE buffer containing lysozyme (final concentration 0.1 mg/mL) and RNase A (ITW Reagents, Barcelona, Spain) at a final concentration 0.1 mg/mL, incubated for 25 min at 37 °C. Proteinase K (VWR Chemicals, Aurora, OH, USA) at a final concentration of 0.1 mg/mL and SDS (Sigma-Aldrich, St. Louis, MI, USA) at a final concentration of 0.5% v/v were added and incubated for 5 min at 65 °C. Genomic DNA was purified using an equal volume of SPRI beads and resuspended in EB buffer (Qiagen, Hilden, Germany).

DNA was quantified with the Quant-iT dsDNA HS kit (ThermoFisher Scientific, Waltham, MA, USA) assay in an Eppendorf AF2200 plate reader (Eppendorf UK Ltd., Stevenage, UK). Extracted DNA was eluted in 10 mM Tris-HCl pH 8.0 or nuclease free water and sent to MicrobesNG for sequencing.

### 4.5. Illumina Sequencing

Genomic DNA libraries were prepared using the Nextera XT Library Prep Kit (Illumina, San Diego, CA, USA) following the manufacturer’s protocol with the following modifications: input DNA was increased 2-fold, and PCR elongation time increased to 45 s. DNA quantification and library preparation were carried out on a Hamilton Microlab STAR automated liquid handling system (Hamilton Bonaduz AG, Bonaduz, Switzerland). Pooled libraries were quantified using the Kapa Biosystems Library Quantification Kit for Illumina. Libraries were sequenced using Illumina sequencers (HiSeq/NovaSeq) using a 250 bp paired-end protocol.

### 4.6. Genome Quality Check and Assembly

Raw reads quality was assessed using FastQC version 0.7.2 [51]. Sequencing adapters of raw fastq data were trimmed using Trimmomatic version 0.38.1 [52] with a sliding window quality cutoff of Phred score Q15 [53]. Cleaned reads were de novo assembled using SPAdes version 3.12.0 [54,55] and contigs were annotated using Prokka 1.11 [56]. All these tools were run using Galaxy|Europe (https://usegalaxy.eu/; [accessed on 15 October 2021]).

### 4.7. Bacterial Speciation and Multilocus Sequence Typing

Species identification and multilocus sequence typing of CoNS sequences were performed using Speciator and MLST tools, respectively; both are part of Pathogenwatch platform (https://pathogen.watch/ [accessed on 15 September 2021]).

### 4.8. Antimicrobial Resistance Genes Identification and Virulence Genes Annotation

Staramr version 0.7.2 [57] was used to search for antimicrobial resistance (AMR)-conferring genes. Prokka version 1.14.6 [56], with the help of “Advanced cut” and “Select line that matches an expression” tools, was used to annotate virulence genes. All these tools were run using Galaxy|Europe (https://usegalaxy.eu/; [accessed on 15 October 2021]).

### 4.9. Statistical Data Analysis

All the data were imported into STATA software version 13.0 (College Station, TX, USA) for analysis where categorical variables (species, sequence types, virulence genes, and antimicrobial resistance genes) were presented as frequencies and percentages.

### 4.10. Ethical Consideration

The HATUA project received research and ethical approval from various regulatory bodies within and outside Tanzania [50]. This sub-study further received approval from the Joint CUHAS/BMC Research Ethics and Review Committee (CRE/490/2021).

## 5. Conclusions

*S. haemolyticus* and *S. epidermidis* harboring *ica*C virulence genes and *dfr*G, *bla*Z, and *mec*A AMR genes were the predominant CoNS causing UTI in Tanzania. New ST of *S. haemolyticus* and *S. epidermidis* were most frequently detected indicating population structure geographical variation of *S. haemolyticus* and *S. epidermidis*. Laboratories should carefully interpret the significant bacteriuria due to CoNS in relation to UTI symptoms and pyuria before labeling them as contaminants, as they may be potential UTI pathogens. Further follow-up studies to document the outcome of the treated patients is needed to add more evidence that CoNS are probable UTI pathogens.

## Figures and Tables

**Table 1:** Distribution of sequence types (ST) per CoNS species.

Species	ST	Number
*S. haemolyticus*	30	8
	56	2
	1	2
	49	2
	145 *	2
	38	1
	66	1
	138 *	1
	139 *	1
	140 *	1
	141 *	1
	142 *	1
	143 *	1
	144 *	1
*S. epidermidis*	490	7
	329	1
	150	1
	1184 *	1
	1185 *	1
	1186 *	1
	1187 *	1
	1188 *	1
	1189 *	1
	1190 *	1
	Unassigned ST	8
*S. simulans*	56	1
*S. lugdunensis*	3	1

* New ST from this study.

**Table 2:** Types and frequency of virulence genes.

Gene	Product	CoNS Species	Frequency (%)
*fbe*	Fibrinogen binding protein	*S. haemolyticus*, *S. epidermidis*, *S. simulans*	13 (12.9)
*icaB*	Poly-beta-1, 6, N-acetyl-D-glucosamine deacetylase	*S. haemolyticus*, *S. epidermidis*, *S. simulans*	13 (12.9)
*icaD*	Poly-beta-1,6-N-acetyl-D-glucosamine synthesis protein IcaD	*S. haemolyticus*, *S. epidermidis*, *S. simulans*	14 (13.9)
*icaA*	Poly-beta-1, 6, N-acetyl-D-glucosamine synthase	*S. haemolyticus*, *S. epidermidis*, *S. simulans*	14 (13.9)
*icaC*	Poly-beta-1, 6, N-acetyl-D-glucosamine export protein	*S. haemolyticus*, *S. epidermidis*, *S. hominis*, *S. cohnii*	47 (46.5)
**Gene(s) singly or in combination**			
*fbe*		*S. haemolyticus (1)*	2 (3.4)
		*S. epidermidis (1)*	
*ica*C		*S. epidermidis (8)*, *S. haemolyticus (19)*	34 (57.6)
		*S. saprophyticus (5)*, *S. hominis (1)*, *S. cohnii (1)*	
*ica*C*, fbe*		*S. haemolyticus (1)*	1 (1.7)
*ica*A*, ica*B*, fbe*		*S. epidermidis (1)*	1 (1.7)
*ica*A*, ica*C*, ica*D		*S. haemolyticus (1)*	1 (1.7)
*ica*A*, ica*B*, icaC, ica*D		*S. epidermidis (5)*	5 (8.5)
		*S. simulans (1)*	
*ica*A*, ica*B*, ica*C*, ica*D*, fbe*		*S. epidermidis (9)*, *S. haemolyticus (5)*	15 (25.4)
**Total**			**59 (100.0)**

**Table 3:** Distribution of AMR genes and their predicted AMR phenoty.pe.

CoNS Species	Genes [Predicted AMR Phenotype]													
	*dfr*G [SXT]	*bla*Z [AMP]	*mec*A [MET]	*aac (6’) aph (2”)* [CN]	*tet*K [TET]	*msr*A [E/AZT]	*erm*(C) [E/AZT]	*mph*C [E]	*fos*B [FO]	*cat(pc221)* [C]	*tet*M [TET]	*tet*L [TET]	*cat(pc233)* [C]	*cat* [C]
*S. haemolyticus (27)*	24	21	21	18	8	11	8	6		4	7	7	3	2
*S. epidermidis (23)*	22	7	1	2	8	5	3	2	12	4			1	
*S. saprophyticus (2)*	2	2	1	1	1	2		1		1				
*S. hominis (4)*	2	2	2	1	2	2								
*S. warneri (2)*	2		1	2	2	1	1				1			
*S. cohnii (1)*	1						1							
*S. simulans (1)*							1							
**Total**	**53**	**32**	**26**	**24**	**21**	**21**	**14**	**9**	**12**	**9**	**8**	**7**	**4**	**2**

Less frequently occurring genes, i.e., str (streptomycin) and msrD, 1 gene each, and cat (chloramphenicol), 2 genes, harbored by *S. haemolyticus*; and aadD (amikacin, tobramycin) and fusB (Fusidic acid), 1 gene each harbored by *S. epidermidis*. **SXT** = sulphamethoxazole/trimethoprim, **AMP** = ampicillin, **MET** = methicillin, **CN** = gentamicin, **TET** = tetracycline, **E/AZT** = erythromycin/azithromycin, **C** = chloramphenicol, **FO**=Fosfomycin, **E**=erythromycin.

## Data Availability

The datasets used and/or analyzed during the current study available on the request from the Director of Research and Innovation, Catholic University of Health and Allied Sciences (vc@bugando.ac.tz). The genomes have been deposited in the European Nucleotide Archive (ENA) with project ID: PRJEB52104.

## Supplementary Materials

The following supporting information can be downloaded at: https://www.mdpi.com/article/10.3390/pathogens12020180/s1, File S1: Sequences of novel alleles detected.
