# Supplementary material for: Molecular Characterizations of the Coagulase-Negative Staphylococci Species Causing Urinary Tract Infection in Tanzania: A Laboratory-Based Cross-Sectional Study
Source: Pathogens. 2023 Jan 24;12(2):180. doi: 10.3390/pathogens12020180 (PMC9967252; doi:10.3390/pathogens12020180)
Supplement: Supplementary file 1 [file pathogens-12-00180-s001.zip › pathogens-2137669-supplementary.pdf]

Supplementary file S1: Sequences of novel alleles detected

>Isolate number TZ010858, TZ013945 and TZ010158

?arcC_8

```
template    TTCTACACTAAAGAAGAAGCAAATCGTATTCAACAGGAAAAAGGTTATCAATTTGTAGAA
query       TTCTACACTAAAGAAGAAGCAAATCGTATTCAACAGGAAAAAGGTTATCAATTTGTAGAA

template    GATGCTGGTCGAGGTTACCGTCGCGTCGTACCATCACCACAACCAATATCTATTATCGAA
query       GATGCTGGTCGAGGTTACCGTCGCGTCGTACCATCACCACAACCAATATCTATTATCGAA

template    CTGGAAAGTATTAAAACTCTAGTAGAAAATGACACACTTGTCATCGCTGCAGGTGGAGGT
query       CTGGAAAGTATTAAAACTCTAGTAGAAAATGACACACTTGTCATCGCTGCAGGTGGAGGT

template    GGTATACCAGTCATTCGCGAACAGCATGATAGCTTTAAAGGTATAGATGCCGTCATCGAT
query       GGTATACCAGTCATTCGCGAACAGCATGATAGCTTTAAAGGTATAGATGCCGTCATCGAT

template    AAAGACAAAACAAGTGCATTATTAGGTGCTGATATTCACTGTGATCAACTCATTATTTTA
query       AAAGACAAAACAAGTGCATTATTAGGTGCTGACATTCACTGTGATCAACTCATTATTTTA

template    ACAGCGATTGATTATGTTTATATCAACTATCATACTGACCAACAACAAGCACTTAAAACA
query       ACAGCGATTGATTATGTTTATATCAACTATCATACTGACCAACAACAAGCACTTAAAACA

template    ACAAATATAGATACGCTTAAAACATATATTGAAGAAGAACAATTCGCCAAAGGCAGCATG
query       ACAAATATAGATACACTTAAAACATATATTGAAGAAGACCAATTCGCCAAAGGCAGCATG

template    CTACCTAAAATCGAATCTGCCATCTCCTTTATTGAAAATAATCCT
query       CTACCTAAAATCGAATCTGCCATCTCCTTTATTGAAAATAATCCT
```

Isolate 10865 and 10924

?arcC_28

```
template    TTCTACACTAAAGAAGAAGCTCATCGTATTCAACAGGAAAAAGGTTATCAATTTGTAGAA
query       TTCTACACTAAAGAAGAAGCTCATCGTATTCAACAGGAAAAAGGTTATCAATTTGTAGAA

template    GATGCTGGTCGAGGTTACCGTCGCGTCGTACCATCACCACAACCAATATCTATTATCGAA
query       GATGCTGGTCGAGGTTACCGTCGCGTCGTACCATCACCACAACCAATATCTATTATCGAA

template    CTGAAAAGTATTAAAACTCTAATAGAAAATGACACACTTGTCATCGCTGCAGGTGGAGGT
query       CTGAAAAGTATTAAAACTCTAATAGAAAATGACACACTTGTCATCGCTGCAGGTGGAGGT

template    GGTATACCAGTCATTCGTGAGCAGCACGATAGCTTTAAAGGTATAGATGCCGTCATTGAT
query       GGTATACCAGTCATTCGTGAGCAGCACGATAGCTTTAAAGGTATAGATGCCGTCATTGAT

template    AAAGACAAAACAAGTGCATTATTAGGTGCTGATATTCATTGTGATCAACTCATTATTTTA
query       AAAGACAAAACAAGTGCATTATTAGGTGCTGATATTCATTGTGATCAACTCATTATTTTA

template    ACAGCGATTGATTATGTTTATATCAACTATCATACTGACCAACAGCAAGCACTTAAAACA
query       ACAGCGATTGATTATGTTTATATCAACTATCATACTGACCAACAACAAGCTCTTAAAACA

template    ACAAATATAGATACGCTTAAAACATATATTGAAGAAGAACAATTCGCCAAAGGCAGCATG
query       ACAAATATAGATACGCTTAAAACATATATTGAAGAAGAACAATTCGCCAAAGGCAGCATG

template    CTACCTAAAATTGAATCTGCCATCTCCTTTATTGAAAATAATCCT
query       CTACCTAAAATTGAATCTGCCATCTCCTTTATTGAAAATAATCCT
```

>Isolate 13945

?tpiA_10

```
template    AATTGGAGCACAAAACGCTTACTTTGAAGAAAGCGGTGCTTATACTGGAGAAACTTCACC
query       AATTGGAGCACAAAACGCTTACTTTGAAGAAAGCGGTGCTTATACTGGAGAAACTTCACC

template    AGTTGCATTATCTGAATTAGGTGTTAAATATGTAGTGATTGGTCACTCAGAGCGTCGTGA
query       AGTTGCATTATCTGAATTAGGTGTTAAATATGTAGTGATTGGTCACTCAGAGCGTCGTGA

template    CTATTTCCACGAAACTGACGAAGAAGTAAACAAAAAAGCGCATGCTATCTTCAATCACGG
query       CTATTTCCACGAAACTGACGAAGAAGTAAACAAAAAAGCGCATGCTATCTTCAATCACGG

template    TATGACACCTATTATTTGTGTAGGTGAATCTGATGAAGAACGTGAAGCTGGTAAAGCAAA
query       TATGACACCTATTATTTGTGTAGGTGAATCTGATGAAGAACGTGAAGCTGGTAAAGCAAA

template    TGAAATCGTAGGCAATCAAGTTAAAAAAGCTGTCGAAGGTTTATCAGATGATCAACTTAA
query       TGAAATCGTAGGCAATCAAGTTAAAAAAGCTGTCGAAGGTTTATCAGATGATCAACTTAA

template    AGAAGTTGTTATTGCATATGAACCAATTTGGGCTATCGGTACTGGTAAGTCATCTACATC
query       AGAAGTTATTATTGCATATGAACCAATTTGGGCTATCGGTACTGGTAAGTCATCTACATC

template    TGAAGATGCAAATGAAATGTGTGCTCACGTACGTCAAACATTAGCTGACTTATCTAGTCA
query       TGAAGATGCAAATGAAATGTGTGCTCACGTACGTCAAACATTAGCTGACTTATCTAGTCA

template    AGAT
query       AGAT
```

?tpiA_57

```
template    AATTGGAGCACAAAACGCTTACTTTGAAGAAAGCGGTGCTTATACTGGAGAAACTTCACC
query       AATTGGAGCACAAAACGCTTACTTTGAAGAAAGCGGTGCTTATACTGGAGAAACTTCACC

template    AGTTGCATTATCTGAATTAGGTGTTAAATATGTAGTGATTGGTCACTCAGAGCGTCGTGA
query       AGTTGCATTATCTGAATTAGGTGTTAAATATGTAGTGATTGGTCACTCAGAGCGTCGTGA

template    CTATTTCCACGAAACTGACGAAGAAGTAAACAAAAAAGCGCATGCTATCTTCAATCACGG
query       CTATTTCCACGAAACTGACGAAGAAGTAAACAAAAAAGCGCATGCTATCTTCAATCACGG

template    TATGACACCTATTATTTGTGTAGGTGAATCTGATGAAGAACGTGAAGCTGGTAAAGCAAA
query       TATGACACCTATTATTTGTGTAGGTGAATCTGATGAAGAACGTGAAGCTGGTAAAGCAAA

template    TGAAATAGTAGGTAATCAAGTGAAAAAAGCTGTCGAAGGTTTATCAGATGATCAACTTAA
query       TGAAATCGTAGGTAATCAAGTGAAAAAAGCTGTCGAAGGTTTATCAGATGATCAACTTAA

template    AGAAGTTGTTATTGCATATGAACCAATTTGGGCTATCGGTACTGGTAAGTCATCTACATC
query       AGAAGTTGTTATTGCATATGAACCAATTTGGGCTATCGGTACTGGTAAGTCATCTACATC

template    TGAAGATGCAAATGAAATGTGTGCTCACGTACGTCAAACATTAGCTGACTTATCTAGTCA
query       TGAAGATGCAAATGAAATGTGTGCTCACGTACGTCAAACATTGGCTGACTTATCTAGTCA

template    AGAT
query       AGAT
```

Isolate 13945 and 626

?mutS_23

```
template   TTTGAAATGAGTTACTTTGAGTTCTCCTGTAGAAACATCACAATAGCATAAACCAAATTC
query      TTTGAAATGAGTTACTTTGAGTTCTCCTGTAGAAACATCACAATAGCATAAACCAAATTC

template   TTCATTTTCGATAAAGCTTAAAATATAATTATTTTTCTTTTCATCCATACCATTTTGATC
query      TTCATTTTCGATAAAGCTTAAAATATAATTATTTTTCTTTTCATCCATACCGTTTTGATC

template   CATAACAGTTCCTGGTGTGACGATTCTTACAACTTCTCTTCTAACCATTCCTTTTGTTTG
query      CATAACAGTTCCTGGTGTGACGATTCTTACAACTTCTCTTCTAACCATTCCTTTTGTTTG

template   CTTTGGATCTTCCATTTGTTCACATATAGCGACCTTATAACCATTATTAATCAATGTTTC
query      CTTTGGATCTTCCATTTGTTCACATATAGCGACCTTATAACCATTATTAATCAATGTTTC

template   AATGTAATTATCAGCAGAATGATATGGTACGCCACACATCGGAATAGGATTTTCTTTTTT
query      AATGTAATTATCAGCAGAATGATATGGTACGCCACACATCGGAATAGGATTTTCTTTTTT

template   AGCATCTCTTTTCGTCAATGTTATTTCAAGTACTCTTGATGCTTCTTTAGCATCATCAAA
query      AGCATCTCTTTTCGTCAATGTTATTTCAAGTACTCTTGATGCTTCTTTAGCATCATCAAA

template   GAACATTTCATAGAAATCTCCGAGTCTAAAAAATAGCAAACAATCATCATAT
query      GAACATTTCATAGAAATCTCCGAGTCTAAAAAATAGCAAACAATCATCATAT
```

Isolate TZ010838

?aroE_24

```
template    GCGGTAAATACAGTTTTAGTTAAAGATGGTAAGTGGATTGGTTATAATACTGATGGAATT
query       GCGGTAAATACAGTTTTAGTTAAAGATGGTAAGTGGATTGGTTATAATACTGATGGAATT

template    GGTTATGTTAATGGTTTAAAACAAATATATGAAGGTATAGAAGACGCTTATATATTAATT
query       GGTTATGTTAATGGTTTAAAACAAATATATGAAGGTATAGAAGACGCTTATATATTAATT

template    TTAGGTGCAGGTGGAGCAAGTAAAGGTATAGCAAATGAATTATATAAAATCGTTCGACCG
query       TTAGGTGCAGGTGGAGCAAGTAAAGGTATAGCAAATGAATTATATAAAATCGTTCGACCG

template    ACTTTAACAGTTGCAAATAGAACGATGTCTCGTTTTAATAATTGGTCGTTAAATATTAAC
query       ACTTTAACAGTTGCAAATAGAACGATGTCTCGTTTCAATAATTGGTCGTTAAATATTAAC

template    AAAATAAATTTAAGCCATGCAGAACGCCATTTAGATGAATTTGATATTATAATAAACACT
query       AAAATAAATTTAAGCCATGCAGAACGCCATTTAGATGAATTTGATATTATAATAAACACT

template    ACACCTGCTGGTATGAACGGCAATACAGATTCTGTAATTTCTTTAAATCGTTTAGCTTCA
query       ACACCTGCTGGTATGAACGGCAATACAGATTCTGTAATTTCTTTAAATCGTTTAGCTTCA

template    CATACTTTAGTAAGTGATATTGTTTATAATCCATATAAAACACCAATACTAATAGAAGCT
query       CATACTTTAGTAAGTGATATTGTTTATAATCCATATAAAACACCAATACTAATAGAAGCT
```

?pyrR_49

```
template    CGTACAATTACACGAATTGCTCATGAAATTCTAGAATATAACAAGGGAACCAAAGATTTA
query       CGTACAATTACACGAATTGCTCATGAAATTCTAGAATATAACAAGGGAACCAAAGATTTA

template    GTTCTATTAGGCATTAAAACAAGGGGTGCTTTTTTAGCACATCGTATACAAGATAAAATA
query       GTTCTATTAGGCATTAAAACAAGGGGTGCTTTTTTAGCACATCGTATACAAGATAAAATA

template    AATTCAATTGAACAACAATTAGTACCAACAGGTACTATCGATATCACGCATTTTCGAGAT
query       AATTCAATTGAACAACAATTAGTACCAACAGGTACTATCGATATCACGCATTTTCGAGAT

template    GACGTTGATAAGGTAGTACAACAAGCTGATCAACGAGCTTTTGATATTAATGTAGATATT
query       GACGTTGATAAGGTAGTGCAACAAGCTGATCAACGAGCTTTTGATATTAATGTAGATATT

template    AATAACAAAGTGGTTGTTATCATTGACGATGTTTTGTATACCGGACGTACAGTAAGAGCC
query       AATAACAAAGTGGTTGTTATCATTGACGATGTTTTGTATACCGGACGTACAGTAAGAGCC

template    TCATTAGATGCGATTTTATTACATACAAGACCTATTAAAATAGGGCTTGCAGCACTTGTG
query       TCATTAGATGCGATTTTATTACATACAAGACCTATTAAAATAGGGCTTGCAGCACTTGTG

template    GATCGTGGTCATCGTGAACTCCCTATACGCGCTGATTTTGTAGGAAAAAATATACCTACA
query       GATCGTGGTCATCGTGAACTCCCTATACGCGCTGATTTTGTAGGAAAAAATATACCTACA

template    GCACGAGA
query       GCACGAGA
```

Staphylococcus haemolyticus

TZ014667.fasta

?arcC_14

```
template   GGCTTATTCTATACAAAAGAACAAGCGGAACAAACAATGGAAGAAAAAGGTTACAAATTC
query      GGCTTATTCTATACAAAAGAACAAGCGGAACAAACAATGGAAGAAAAAGGTTACAAATTC

template   GTAGAAGACTCTGGACGAGGTTATCGTCGTGTTGTACCTTCTCCAATGCCAATCAACATT
query      GTAGAAGACTCTGGACGAGGTTATCGTCGTGTTGTACCTTCTCCAATGCCAATCAACATT

template   GTAGAACTAGATAGCATTGAAACATTAATTAAACACGGTACATTAGTTATTGCAGCTGGT
query      GTAGAACTAGATAGCATTGAAACATTAATTAAACACGGTACATTAGTTATTGCAGCTGGT

template   GGTGGCGGTATCCCAGTTGTTAAAAAAGAAGGTAACTATAAAGGCGTCGATGCTGTTATC
query      GGTGGCGGTATCCCAGTTGTTAAAAAAGAAGGTAACTATAAAGGCGTCGATGCTGTTATC

template   GATAAAGATAAAACAAGCGCTTTACTTGCGGCACACTTAAAATCAGATCAATTAATTATC
query      GATAAAGATAAAACAAGCGCTTTACTTGCGGCACACTTAAAATCAAATCAATTAATTATC

template   TTAACAGCAGTAGATTATGTTTATATTAATTACGGTAAAGATAATCAAGAAGCTTTAGGT
query      TTAACAGCAGTAGATTATGTTTATATTAATTACGGTAAAGATAATCAAGAAGCTTTAGGT

template   GAAGTAACAGTGGATGAAATGAATCAACATATTGCTGACGGT
query      GAAGTAACAGTGGATGAAATGAATCAACATATTGCTGACGGT
```
